# On the Use of Chloroform

**Published:** 1851-02

**Authors:** 


*On the Use of Chloroform.* By J. D. Gross, M. D.

I have used chloroform extensively during the last two years, and am disposed to believe that, as an anæsthetic agent, it is, in every respect, preferable to sulphuric ether. The first case in which I exhibited it was that of a colored woman who had an enormous tumor of the thigh, involving the femoral artery. The operation was performed in February, 1848, and, although necessarily tedious, was unaccompanied by the slightest pain. The patient soon fell into a tranquil sleep, and so continued during the dissection. Since that period, I have employed chloroform constantly in all my most important operations, and have never, except in one solitary instance, witnessed any disagreeable effects from it. The case to which I refer was that of Benjamin Cunningham, of Western Virginia, sixteen years of age, who had a large tumor on the left side of the neck. He was tall and slender, and of a feeble, delicate constitution. The operation was performed before the medical class of the University of Louisville, in November, 1849, and lasted less than five minutes. The tumor weighed nearly six pounds. The quantity of chloroform administered was very small, not certainly exceeding one drachm and a half; it was inhaled from a small cup-shaped sponge. The patient became affected in a few seconds, falling into a profound sleep, and thus continuing for fifty-five minutes. The pulse during the whole of this time was small and feeble, but perfectly distinct; the countenance was pale; the eyes were turned up and the pupils dilated; and the extremities were cold. There was no vomiting, and apparently no nausea. As soon as the excision of the tumor was over, and several small vessels tied, the youth was removed from the amphitheatre into my private room, where, under the influence of cold air, of volatile alkali freely applied to the nose, of sinapisms to the præcordial region, and of warmth to the feet, he gradually awoke to consciousness. It may be observed here that the utmost care was taken, during the performance of the operation, to prevent the entrance of air into the veins of the neck. Had this precaution been neglected, it might have been difficult to determine whether the effect in question was produced by this cause alone, or partly by it and partly by the chloroform. Cunningham rapidly recovered from the operation without an untoward symptom.

This is the only instance, out of several hundred, in which the use of chloroform at all alarmed me. For a time it was uncertain how the case would terminate; and I must confess I felt greatly relieved when I saw the young gentleman regain his consciousness.

I have administered chloroform at almost every age, from that of sixteen months up to that of seventy years; in both sexes; in slight operations as well as the most severe; and under various circumstances of health and disease. In some of my cases, it has been found necessary to keep up a sustained action of the remedy for nearly half an hour.

I have remarked that there is much diversity in different individuals in relation to the susceptibility of the system to chloroform. In some, as in young Cunningham, the smallest quantity sufficed to produce the desired effect; while in others a large dose was necessary, as well as a longer exhibition of the remedy. Children usually require very little, much less, comparatively speaking, than grown persons, and in them the effects also seem to be less evanescent.

I have not noticed in any of my cases that the use of chloroform exerted any injurious effect upon the recovery of my patients; that it excited an unusual degree of fever or local irritation; or that it retarded, impeded, or prevented the adhesive process. I have repeatedly witnessed the occurrence of erysipelas after it, but not more frequently, I think, than would have taken place had the operation been performed without it. The excitement consequent upon the administration of the remedy has usually passed off in a short time, and in no instance have I witnessed any secondary effects that were justly ascribable to its influence.

In several of my cases, transient opisthotonos appeared to have been produced by the inhalation of tnis article. This phenomenon was strikingly displayed in a young gentleman of Covington, Ky., to whose hip I applied the actual bautery on account of coxalgia. While under the influence of chloroform, which he inhaled rather badly, his head was forcibly thrown backwards, and the body forwards, precisely as in opisthotonos. The effect did not last longer than fifteen or twenty seconds. No stiffness of the jaws was noticed.

On two occasions, while performing the operation of lithotomy, the patients were affected with the most violent priapism, which, however, subsided, in each case, in less than half a minute. The ages of the patients were, respectively, four and twenty-two years. I may remark here that I never witnessed a similar result in any of my stone cases in which chloroform was not administered.

Since I have commenced the use of chloroform, I have abandoned that of sulphuric ether altogether, being persuaded, from ample experience, that it is, in every respect preferable. It produces the desired effect in a much shorter time, causes less excitement of the brain, is more persistent in its action, and is more easily inhaled. In a word, I have every confidence in its efficacy and safety, and I could hardly imagine a more desirable anæsthetic agent.

Finally, my rule always is to bring the system gradually, not rapidly, under the influence of this remedy; an object which is usually attained in thirty or forty seconds, provided the patient gives us his thorough co-operation, and does not embarrass us by his obstinacy or timidity.—*Trans. Am. Med. Association.*

